# Leadership in Public Health: New Competencies for the Future

**DOI:** 10.3389/fpubh.2015.00024

**Published:** 2015-02-26

**Authors:** Nick Yphantides, Steven Escoboza, Nick Macchione

**Affiliations:** ^1^Health and Human Services Agency, County of San Diego, San Diego, CA, USA; ^2^Hospital Association of San Diego and Imperial Counties, San Diego, CA, USA

**Keywords:** leadership, public health leadership, competencies, public health, values, servant leadership

In *Contemporary Public Health*, Keck, Scutchfield, and Holsinger call for changes in vision among those leading the evolution of healthcare from individualistic to community-wide approaches to improve health status (1). Having new vision is necessary but not sufficient; new leadership skills are also needed in order to implement and sustain change. This article summarizes our thoughts about new competencies essential to moving our community’s health system to meet the demands of public health in the twenty-first century.

Since the start of the New Millenium, “public health” has emerged from a behind-the-scenes discipline to a widely recognized leader of community and global initiatives. Concurrently, the healthcare delivery system has embraced “population health” as fundamental to achieving success in its traditional role of caring for individuals (2). These trends have challenged those leading both sectors and created a context that promotes innovations in collaboration. The County of San Diego is an excellent example of the successes and challenges of such collaboration. Those leading new alliances and partnerships have had to be nimble, courageous, and innovative. They have had to learn new content and develop new leadership skills.

San Diego’s health environment has a wealth of proficient leaders and highly skilled managers, as well as strong universities with programs in all clinical professions, public health, and healthcare management and policy. This article combines the insights of three senior leaders on the changes required in our own leadership based on the evolution of the environment, suggests the “competencies” required to deal with those changes, and recommends how these competencies can be taught and/or learned. Our comments are framed in the academic jargon of competencies, include “knowledge, skills, and attitudes,” but recognize that, in the practitioner’s world, these competencies are viewed as practical techniques for orchestrating change and achieving performance. The immediate context of our work is San Diego County, California.

## Background

San Diego County is the fifth most populous county in the United States (US), with an estimated 3.2 million people (3). The most southwest county in the US, San Diego has a population highly diverse in race, language, ethnic heritage, age, education, income, and almost all other demographic characteristics. The County has 18 military installations and 18 federally recognized tribal nations, as well as the world’s busiest international border crossing. Geographically, it is approximately the size of Connecticut. The topography ranges from coast line to mountains. Major educational institutions include the University of California San Diego, San Diego State University, community colleges, and a number of private colleges, many offering education and training for the health professions.

The health system is well developed. San Diego County has seven major integrated health care delivery systems, an array of community clinics, multiple senior housing complexes, numerous community service agencies, and a strong public health department that is a key component of the County of San Diego Health and Human Services Agency (HHSA). Although the healthcare delivery landscape is highly competitive, all hospitals have participated in a joint Community Health Needs Assessment conducted by the Public Health Institute of San Diego State University under the auspices of the Hospital Association of San Diego and Imperial Counties (4).

Four years ago, HHSA launched *Live Well San Diego* (LWSD) (5), a 10-year comprehensive initiative to create a healthy, safe, and thriving County for all 3.2 million residents. Through innovative collaboration among health care, human services, private sector, and community organizations, *LWSD* supports positive healthy choices, pursues policy changes for a healthy environment, and improves the human service culture.

Below, we highlight the evolution of leadership as we and our colleagues have navigated through a complex, changing environment and then use selected programs in LWSD to illustrate the collaboration that is advancing the health of the entire community.

## Essential Attributes of Leadership

Although many theories of leadership have been proposed, we posit that the fundamental attributes of leaders have been constant over time and across continents. Leaders are people with *Vision* – they see a future different than the *status quo*. They have *Influence* to drive change – they are able to communicate their vision and win others over to embrace and implement it. In addition, leaders are grounded in *Values*, which provide a foundation for Vision and a passion to achieve personal and organizational mission. These essentials have characterized leaders for generations, but how they play out in public health continues to evolve.

## Vision

What has changed in public health over the past 15 years? The Patient Protection and Affordable Care Act (ACA) has appropriately been credited with producing major changes in the health system. While its most visible impact has been on coverage and financing, the ACA for the first time created a National Health Strategy and a Prevention and Public Health Fund to help implement it. Several other factors have also contributed to creating an environment that recognizes the essential role of public health, most prominent among them:

- Wide-spread acceptance of the ecological model of health.
- Clinical advances in understanding and managing health.
- Recognition of the impact of behavior on health.
- Technological advances in biomedical and management technologies.
- The Information technology revolution, including electronic health records and “Big Data” analytics.
- Emphasis on a “systems perspective.”

The Vision for a new health system has best been articulated by Berwick and colleagues at the Institute for Healthcare Improvement in the concept of “The Triple Aim:” (6) “Better health for the population. Better care for the individual. At lower per capita cost.” This Vision is concise, expressed in a graphic, and easy to communicate to others. The ACA incorporated all of the Triple Aim concepts. The nation-wide embrace of the Triple Aim has made it possible for leaders in public health to champion an explicit Vision to transform a fragmented system through open communication, consensus building, stakeholder involvement, and processes for collaborative planning at the local level.

## Influence

Influence is essential to achieve wide-spread change. Ideally, it is grounded in knowledge, which can be gained through formal education and expertise, gained through involvement with a broad range of people and institutions, and based upon accomplishments that have brought recognition and respect.

The trends noted above have increased awareness of the value of a systems perspective. The “systems perspective,” broadly defined, has enabled public health to break out of the siloed role in which it had been typically viewed in the US to become interdisciplinary, inter-agency, and inter-organizational. For example, following 9/11, “preparedness” efforts integrated public health with military, fire, transportation, health care institutions, and social service organizations, among other entities. Well before Ebola, the global spread of diseases, such as SARS and HIV/AIDS, created awareness of international networks of public health organizations and the relationship of public health to other public and private sector organizations from transportation agencies to private employers. The “One Health” movement, the “Green” movement, and other recent trends reinforce the notion that public health is an integral player in many private as well as public initiatives and policies.

In short, the sphere of “influence” for public health has broadened immeasurably, creating both the opportunity and the necessity for public health leaders to expand their relationships far beyond their traditional sphere of local and state health departments.

## Values and Competencies

The traditional values of public health include service and interdisciplinary cooperation. The concept of “servant leader” characterizes many leaders of health organizations, including public health. Putting collective wellbeing ahead of personal gain is a priority that today can be measured as well as espoused. The values underpinning the “new” Vision of public health must include a willingness to change, to collaborate, and to be a central player in the health system of the future.

The “Competencies” fundamental to public health leadership include updated Knowledge, Skills, and Attitudes (KSAs). The knowledge base required is much broader than in previous centuries. Leaders must have in-depth understanding of the developments listed above – much broader and more extensive than in the past. In addition, public health leaders must have working familiarity with public policy, strategic planning, information management, social media, managed care, cultural competence, and human resource management, among other topics. Skills include communication with multiple audiences employing new technologies, inter-organizational collaboration, networking abilities, advocacy, and change management. Attitudes include the new values noted above.

## Educators and Learners

One of the challenges of teaching the new generation of public health leaders is that many of those in senior teaching positions in the health professions fields have not themselves acquired the new vision and values. In order to educate a new generation of leaders in public health, the education system needs to change as well as the health system. The recognition of practitioners as experts, an academic appointment system that advances those who practice in the field as well as those who publish, rewards structured to encourage interdisciplinary endeavors, field experiences for students … all will contribute to changing education to produce a new generation of leaders.

The recent explosion of schools and programs specifically focused on the discipline of public health is noteworthy. The more the US has an “educated citizenry,” as recommended by the Institute of Medicine in 2003 (7), the more the nation will achieve a “readiness for change” to improve individual behaviors and social norms that promote health. In addition, the more that public health is recognized as a discipline, the more influence and power public health professionals can be expected to have (8). However, caution is in order. For public health and the healthcare delivery systems to act in concert, education should be structured to reinforce a collaborative approach. Clinicians need to appreciate public health, and public health professionals must work in tandem with clinicians. This collaborative approach among professionals then argues for close working relationships among public health faculty and faculty of clinical disciplines, as well as management and technical disciplines. Public health leaders must have breadth of perspective and influence that exceeds the bounds of any given discipline. Team work and professional recognition can be taught in didactic settings, but they must also be learned and reinforced through field placements, practicums, cross-venue site visits, and a variety of other techniques.

## Live Well San Diego

*Live Well San Diego* exemplifies the ability of a shared vision of “wellness” and a commitment to collaboration to enlist healthcare and other community leaders to collectively improve individual and population health. Among the many efforts being pursued under the *LWSD* umbrella, the following illustrate the approach and the collective impacts that it achieves:

- The San Diego Care Transitions Partnership has brought together 4 health systems (13 hospitals, collectively serving 92% of the Medicare fee for-service population) and HHSA’s Aging and Independence Services to provide the combination of health care and social support services needed to reduce 30-day all-cause hospital readmissions. More than 23,000 patients have been served since the program started in 2013, significantly improving clinical outcomes and achieving demonstrated cost savings for Medicare.
- The Love-Your-Heart Campaign each Valentine’s Day enlists an array of community partners throughout the County, including health care providers, employers, and local fire departments, to measure blood pressure and help to make San Diego a “heart attack and stroke-free zone”. In 2014, almost 18,000 people were screened.
- The Chula Vista Elementary School District under the leadership of the District Superintendent, in partnership with County Public Health Services, and with participation from the entire District staff has introduced healthy eating to students and parents, revamped the physical education curriculum, and reduced students’ body mass index (BMI) by 3.2% over 2 years. This program is now being replicated throughout the County’s 42 school districts.

## Conclusion

The era of the ACA is bringing vision, influence, and leaders’ passion to transform an inadequate medical care delivery system into a more effective health system for the future. Public health is emerging as a trans-disciplinary field that integrates public health concepts and functions with healthcare delivery and clinical care of the individual. The health systems leaders of the future must have a vision that maximizes the contributions of public and individual health, as well as clinical and management approaches. Values that drive the passion to create and continuously improve the health of the nation must include the courage to push for change, an evidence-based approach to decision-making, and the skills to move political, organizational, and individual behavior. To train the future leaders of the health system, the education system must change as well. San Diego County demonstrates that these challenges can indeed be met.

## Conflict of Interest Statement

The authors declare that the research was conducted in the absence of any commercial or financial relationships that could be construed as a potential conflict of interest.
